# Draft Genome Sequences of Two Phylogenetically Distinct Clostridium gasigenes Strains, CM001 and CM004, Isolated from Chilled Vacuum-Packed Meat

**DOI:** 10.1128/MRA.01128-20

**Published:** 2020-10-15

**Authors:** Joseph Wambui, Nicole Cernela, Marc J. A. Stevens, Sabrina Corti, Roger Stephan

**Affiliations:** a Institute for Food Safety and Hygiene, Vetsuisse Faculty, University of Zurich, Zurich, Switzerland; University of Rochester School of Medicine and Dentistry

## Abstract

We present the draft genome sequences of Clostridium gasigenes strains CM001 and CM004. The genomes are 4,147,089 and 4,191,074 bp with GC contents of 28.7% and 28.8%, respectively. Although both strains belong to the same species, whole-genome sequence-based analyses reveal that the strains are phylogenetically distinct.

## ANNOUNCEMENT

Clostridium gasigenes is an anaerobic spore-forming psychrophilic clostridium causing blown-pack spoilage (BPS) in chilled vacuum-packed meat (1), but its genomic data are limited, thus hindering the development of molecular BPS studies in this species. Here, we present the draft genome sequences of *C. gasigenes* strains CM001 and CM004, isolated from meat juice samples of chilled vacuum-packed lamb and veal, respectively (2).

In order to activate and promote spore germination of the isolates, meat juice samples (1 ml) were treated with ethanol (50% [vol/vol]; 1 h; 30°C) and lysozyme (4 mg/ml; 30 min; 37°C). Subsequent isolation steps were carried out anaerobically at 4°C in anaerobic jars containing Oxoid AnaeroGen sachets (Thermo Scientific). The samples were enriched in prereduced peptone-yeast-glucose-starch medium for 3 weeks and then plated onto Columbia agar supplemented with 5% defibrinated sheep blood (CBA) and incubated for 3 weeks. For genomic DNA extraction, a single hemolytic colony from each sample was subcultured on CBA for 2 weeks.

Genomic DNA was isolated using the DNA blood and tissue kit (Qiagen, Hombrechtikon, Switzerland). Sequencing libraries were prepared using Nextera DNA Flex chemistry (Illumina, San Diego, CA, USA). The resulting transposome-based libraries were sequenced on an Illumina MiniSeq sequencer. The sequence reads, which were in the 150- to 300-bp format for both genomes, were 1,092,152 and 1,387,492 bp for CM001 and CM004, respectively. Reads were checked for quality using the software package FastQC 0.11.7 (3) and then assembled using software based on SPAdes 3.0 (4), Shovill 1.0.9 (https://github.com/tseemann/shovill). The assemblies were filtered, retaining contigs greater than 500 bp. The quality of the genomes was determined using CheckM (5), and the genome was annotated using the NCBI Prokaryotic Genome Annotation Pipeline (6) and the RAST pipeline (7). Default parameters were used for all software and Web servers.

The genomes of CM001 and CM004 were assembled into 47 and 64 contigs and comprise 4,147,089 and 4,191,074 bp with GC contents of 28.7% and 28.8%, respectively. The genomes of CM001 and CM004 have 3,955 and 4,045 genes, respectively, out of which 3,862 and 3,951 are coding genes, while 93 and 94 are RNAs, respectively. The genome coverage, contig *N*_50_, and contig *L*_50_ are 40×, 456,201, and 4, respectively, for CM001, compared to 50×, 156,430, and 7, respectively, for CM004.

The 16S rRNA sequences were extracted *in silico* using ContEST16 (8) and used for strain identification in the 16S-based identification tool (8), whereby both strains were identified as *C. gasigenes* and validated using digital DNA-DNA hybridization (dDDH) (9) and average nucleotide identity (ANI) (10). Comparatively, the dDDH value between CM001 and CM004 was 54.1% and ranged between 50.2% and 66.4% when either strain was compared with *C. gasigenes* 8809^T^ or *C. gasigenes* CGAS001 at the subspecies level. The values are below the 79% threshold for subspecies delimitation (9) and confirm a recent study showing genetic diversity among *C. gasigenes* strains (11). Further studies can determine whether the genetic diversity influences the role of *C. gasigenes* in BPS.

### Data availability.

The whole-genome shotgun sequencing data for CM001 and CM004 have been deposited at DDBJ/ENA/GenBank under the accession no. JACKWY000000000 and JACKWV000000000, respectively. The versions described in this paper are JACKWY010000000 and JACKWV010000000, respectively. The raw sequencing reads have been deposited in the SRA under the accession no. SRX8947911 and SRX8947914, respectively.
